# A Pilot Study Evaluating the Effectiveness of Biofeedback-Based Anal Strengthening Exercises in Treating Radiotherapy-Related Faecal Incontinence

**DOI:** 10.7759/cureus.11285

**Published:** 2020-11-01

**Authors:** Benjamin P Scott, Janet Candido, Christopher J Young, Jonathan Hong

**Affiliations:** 1 Colorectal Unit, Royal Prince Alfred Hospital, Sydney, AUS

**Keywords:** faecal incontinence, biofeedback, pelvic radiotherapy

## Abstract

Purpose

Radiotherapy to the pelvis and especially the anal sphincters can result in long-term faecal incontinence. The aim of this study was to assess the effectiveness of biofeedback anal strengthening exercises in radiotherapy-related faecal incontinence.

Methods

A retrospective cohort study was performed on seven patients with radiotherapy-related faecal incontinence. Education and biofeedback based anal strengthening exercises were provided. Baseline and follow-up measurements were performed assessing anal sphincter fatigue time, resting, squeeze, and cough pressure. Continence scores and quality of life measures were assessed. Patients were followed up at five months.

Results

The radiotherapy target varied between prostate, uterus, and rectum. Four of the seven patients were female. Pescatori (0-10) and St Mark’s (0-10) continence scores had a median improvement of 2 (range 0-5) and 1 (range 0-8) respectively. Self-completed patient continence scores (0-10mm) had a median improvement of 2mm (1-6mm). Anal fatigue time measurements (Isotonic Fatigue Time (seconds) and Isometric Fatigue Time (Cycles)) showed a median improvement of three seconds (-4 - 36 seconds) and three seconds (-1 - 6 seconds), respectively. Resting Pressure (mmHg), Squeeze Pressure (mmHg) and Cough Pressure (mmHg) showed median improvement of 10mmHg (-10-21mmHg), 15mmHg (-16 - 100mmHg) and 21mmHg (-3 - 53mmHg), respectively. Patient-defined quality of life (QOL) measures showed a median change of 0.2 (range 0-0.5).

Conclusion

Patients in this pilot study with radiotherapy-related faecal incontinence had improved anal pressure metrics, continence, and QOL following biofeedback based anal strengthening exercises. These early results suggest a benefit for anal strengthening in patients undergoing pelvic radiotherapy.

## Introduction

Faecal incontinence is the involuntary loss of solid or liquid faeces. Individuals with this condition suffer significant embarrassment and morbidity and it places significant burden on carers and health services [1]. In Australia the prevalence of faecal incontinence in women and men is 5.3% and 5.5% respectively [2]; representing over 1 million people. Given the sensitive nature of this condition, it is probably underreported. The risk factors for developing faecal incontinence include advancing age, diarrhoea, urinary incontinence, diabetes, hormone therapy, rectal resection, and pelvic radiation therapy [1]. Damage to normal tissue during radiation therapy can lead to chronic gastrointestinal problems such as incontinence [3]. Radiation induces changes in the normal physiological functions of continence and subclinical disease can be destabilised [4]. Unfortunately, assessment of anorectal function is rarely comprehensive and loss to follow up makes it very difficult to assess [3].

Biofeedback retraining involves the use of mechanical or electrical devices to increase the awareness of a biological response (in this instance, the control of anal sphincter muscles) facilitating patient learning and improved control [5]. It provides patients with visual, biological, and verbal feedback on their ongoing treatment.

Biofeedback retraining is an established treatment modality for faecal incontinence [6] however its role in post-radiotherapy patients for pelvic malignancy is yet to be established. We retrospectively evaluated the effect of biofeedback on patients with faecal incontinence following pelvic radiotherapy.

This project was presented as a poster at the Royal Australasian College of Surgeons 88th Annual Scientific Congress in 2019.

## Materials and methods

Methodology

Patients who were referred for treatment for incontinence after pelvic radiotherapy were retrospectively identified. Patients were included in the study if they had faecal incontinence following radiotherapy and were engaged in the biofeedback retraining program. Participants were excluded if they underwent a surgical procedure that removed the anus (abdominoperineal resection) or they had a de-functioning stoma. 

Biofeedback is a type of reconditioning therapy with the aim of increasing a patient’s conscious control of the pathways controlling defecation. Various modalities have been described including balloon systems to facilitate patient awareness of rectal distension, ultrasound visualisation of the anal sphincter and demonstrating the effects of a patient's exercises and the use of anal manometric probes to teach the patient how to exercise the anal sphincter [7]. Our program is based on the latter and is described below. 

**Figure 1:**
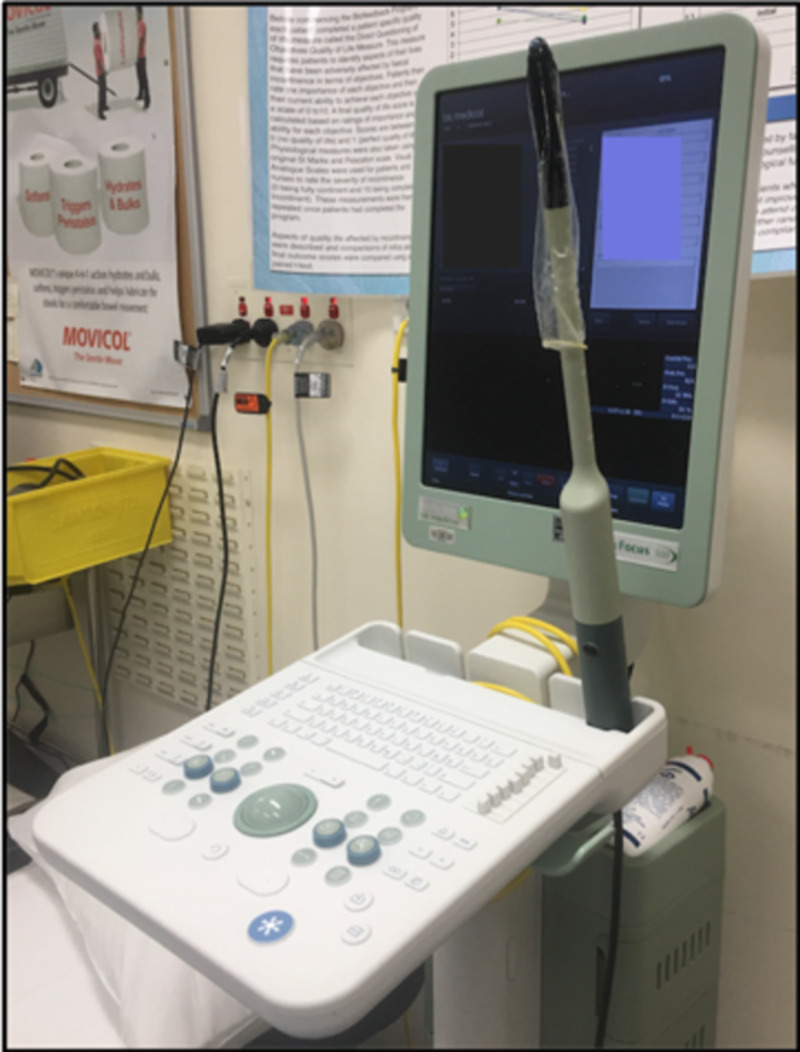
Our Anal Manometer Probe and Display Screen

Our biofeedback retraining program initially takes 90 minutes with education and teaching of anal strengthening exercises. An anal manometer records pressure measurements including resting, cough, and squeeze pressure in addition to the length of time the patient can hold a squeeze without muscle fatigue. This is displayed on a visible screen so the patient receives immediate visual feedback on their actions (Figure 1). This mirrors other studies that have focused on the overall strength and endurance of the anal sphincter [8]. Following this, patients perform the exercises in their own time at regular intervals. Patients who consent to the session have tolerated it well, however some patients are not willing to attempt the program. Quality of life measures, patient-reported continence and objective continence scores are also recorded at this time. The biofeedback program is coordinated by a specialist nurse practitioner who delivers five sessions over four to six months. Our program is robust and has demonstrated effectiveness in more than 70% of patients [9].

Patients in the study received face to face education and biofeedback-based anal strengthening exercises. Baseline and follow-up anal pressure measurements were performed assessing anal sphincter fatigue time, resting, squeeze, and cough pressure. Continence scores (Pescatori and St Mark’s) [10,11] and patient defined quality of life measures were also obtained [12]. Our quality of life measures were defined, by the patient, at the commencement of the program. Patient defined goals included minimising leakage, avoiding soiling and the ability to engage in social activities without fear. A self-completed patient continence score was also completed. The patients were asked to mark a visual analogue scale from 0-10 (10 being complete incontinence) before and after treatment. Visual analogue scales are often used in the evaluation of faecal incontinence and are easily understood by patients [13,14]. Follow-up was completed at four to six months. 

Following approval by our local Human Research Ethics Committee (Research Ethics and Governance Office Royal Prince Alfred Hospital; LNR/18/RPAH/577) the prospectively collected data was retrospectively collated using Research Electronic Data Capture software (REDCap). Statistical analysis was performed using Excel with the Data Analysis Toolpak. Descriptive statistics were calculated including mean, median, standard deviation and range. Given the small sample size, we used non-parametric data tests. Statistical significance of median differences across variables was calculated using the Wilcoxon Matched Pairs Signed Rank Test. The P-value is given as a range, using the critical values in appendix B12 of Zar, as there are fewer than 100 matched pairs [15].

## Results

**Figure 2:**
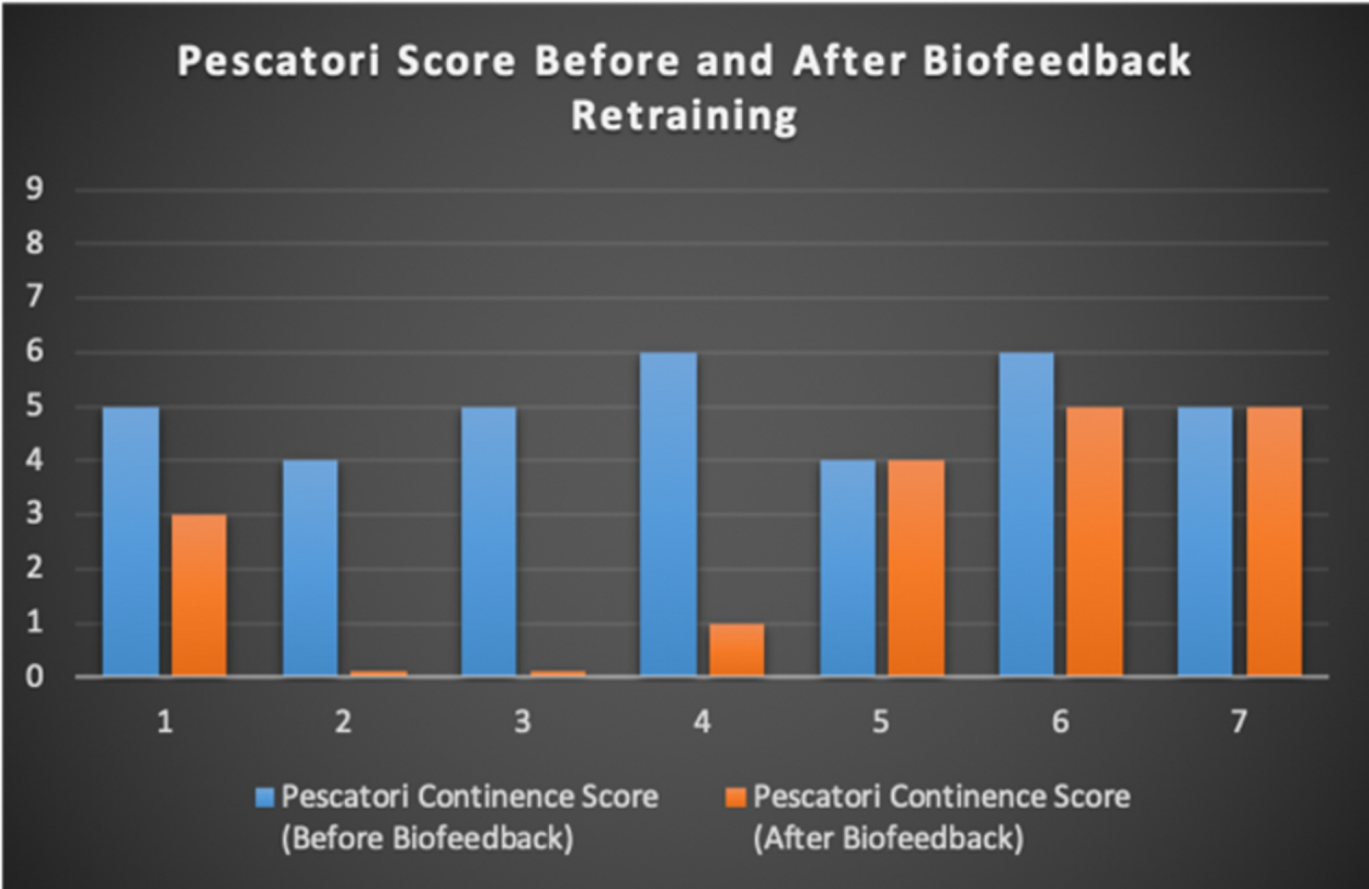
Pescatori Continence Scores For Each Patient Before and After Biofeedback Retraining

**Figure 3:**
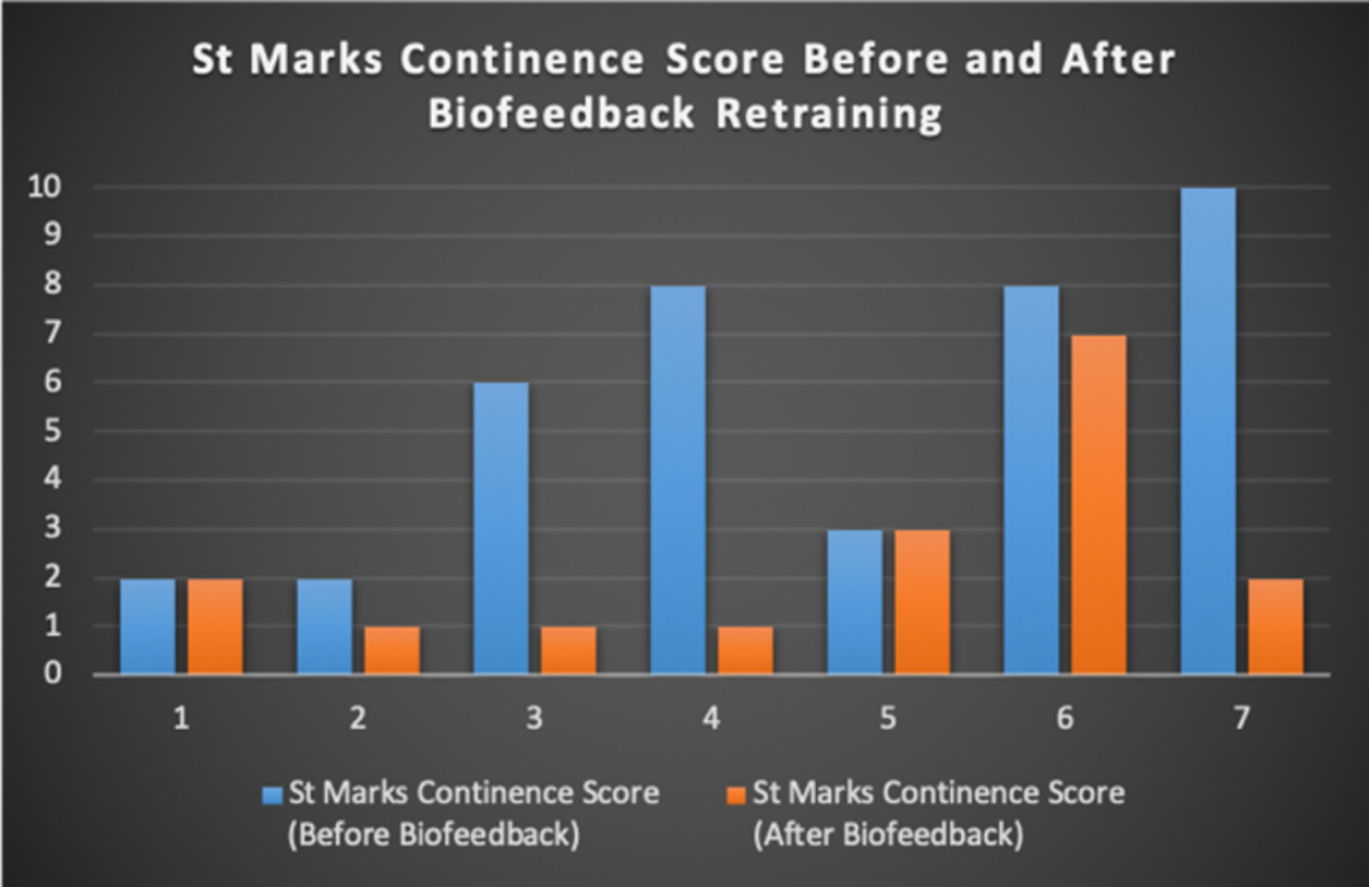
St Marks Continence Scores For Each Patient Before and After Biofeedback Retraining

**Figure 4:**
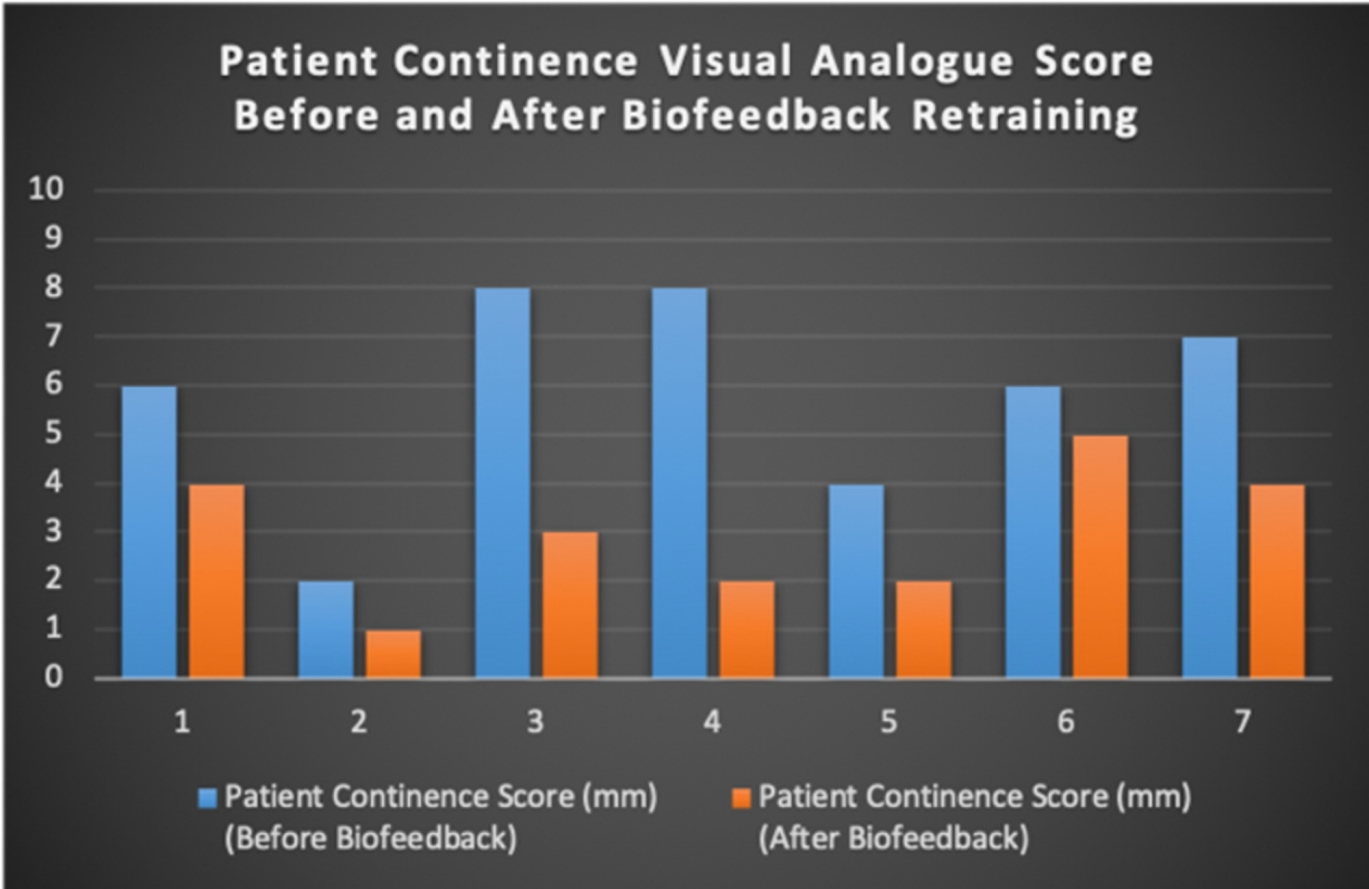
Self Scored Continence Scores For Each Patient Before and After Biofeedback Retraining

**Figure 5:**
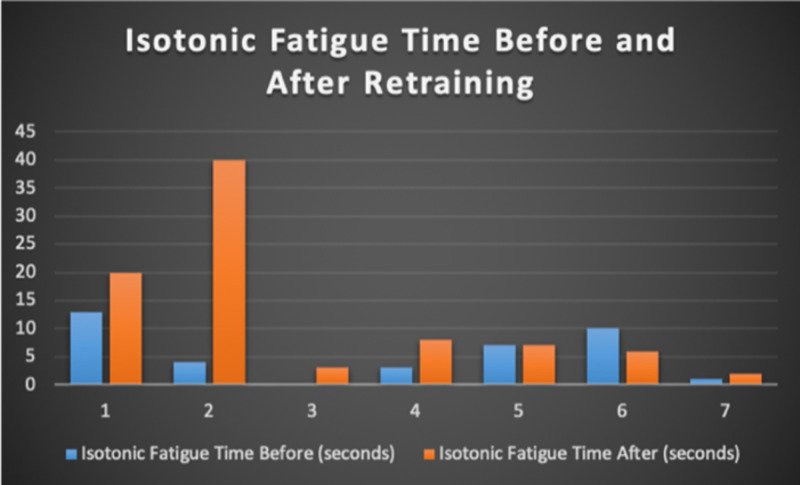
Isotonic Fatigue Time For Each Patient Before and After Biofeedback Retraining

**Figure 6:**
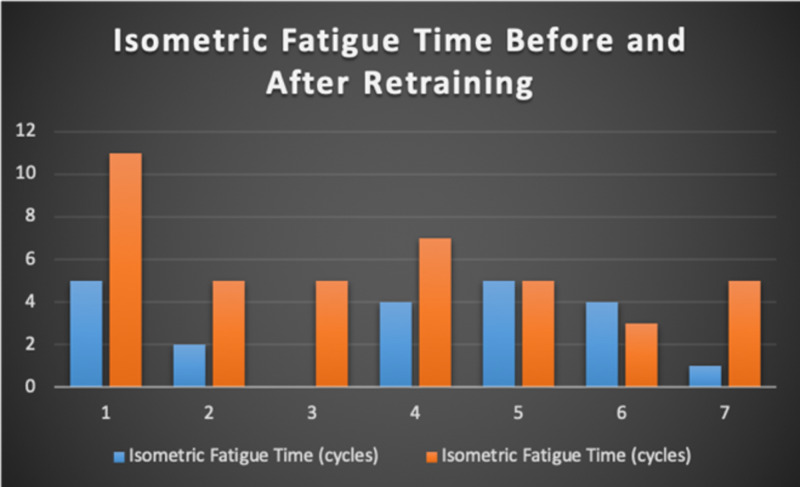
Isometric Fatigue Time For Each Patient Before and After Biofeedback Retraining

**Figure 7:**
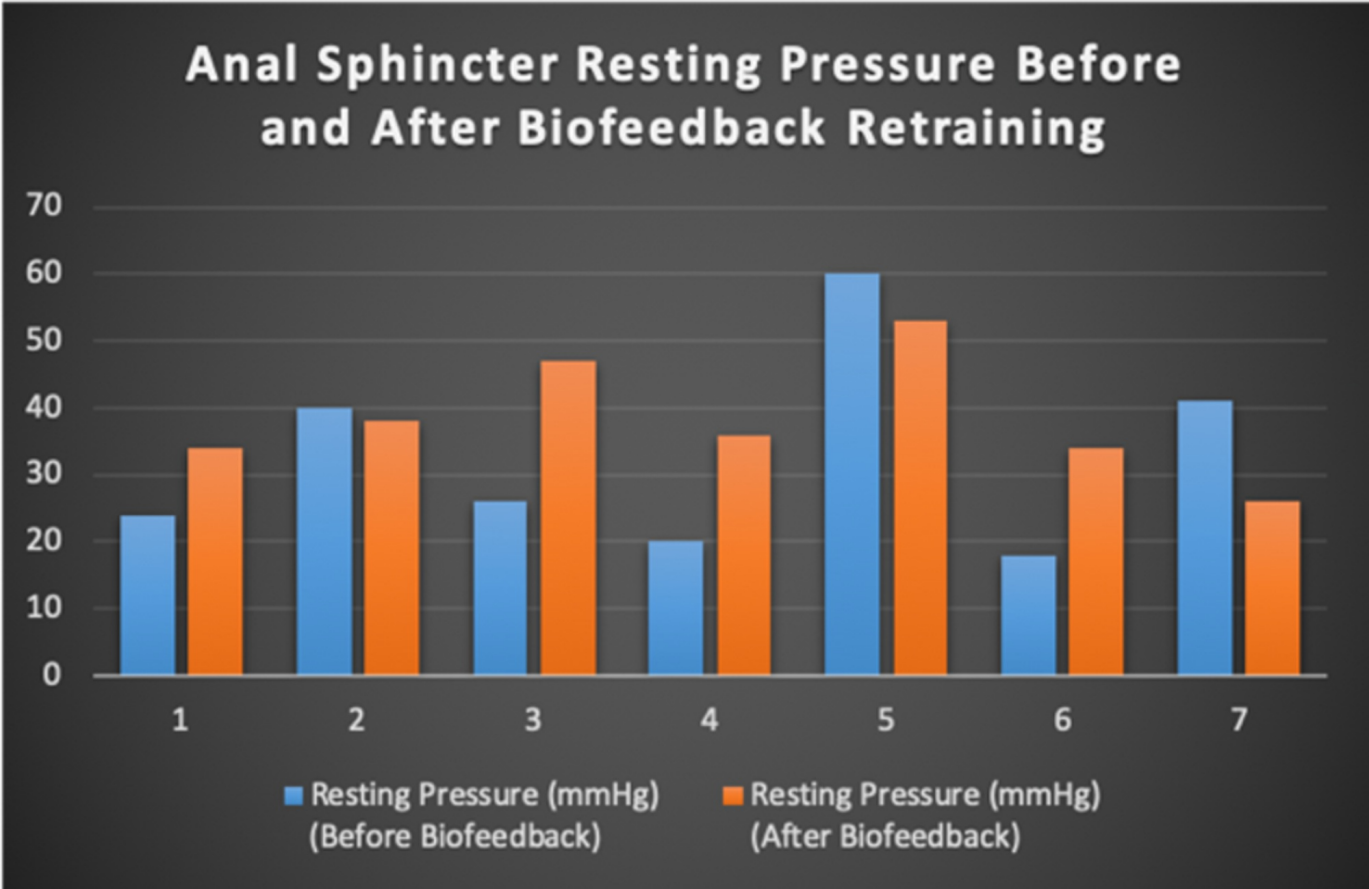
Anal Sphincter Resting Pressure For Each Patient Before and After Biofeedback Retraining

**Figure 8:**
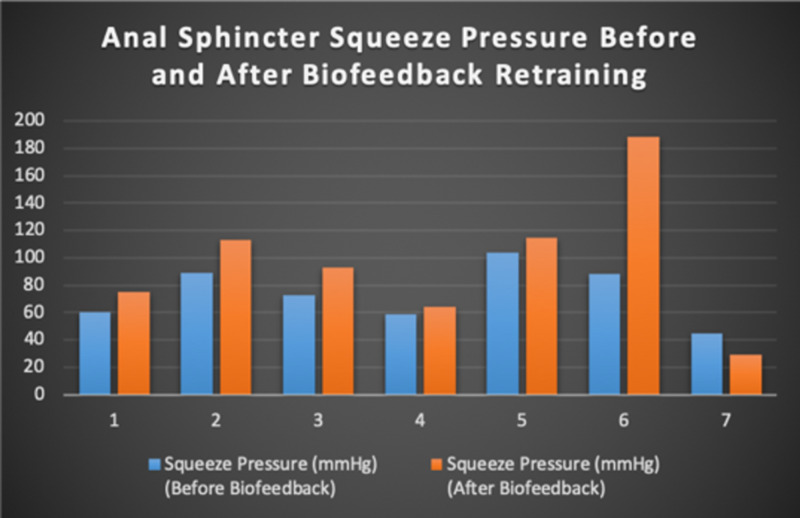
Anal Sphincter Squeeze Pressure For Each Patient Before and After Biofeedback Retraining

**Figure 9:**
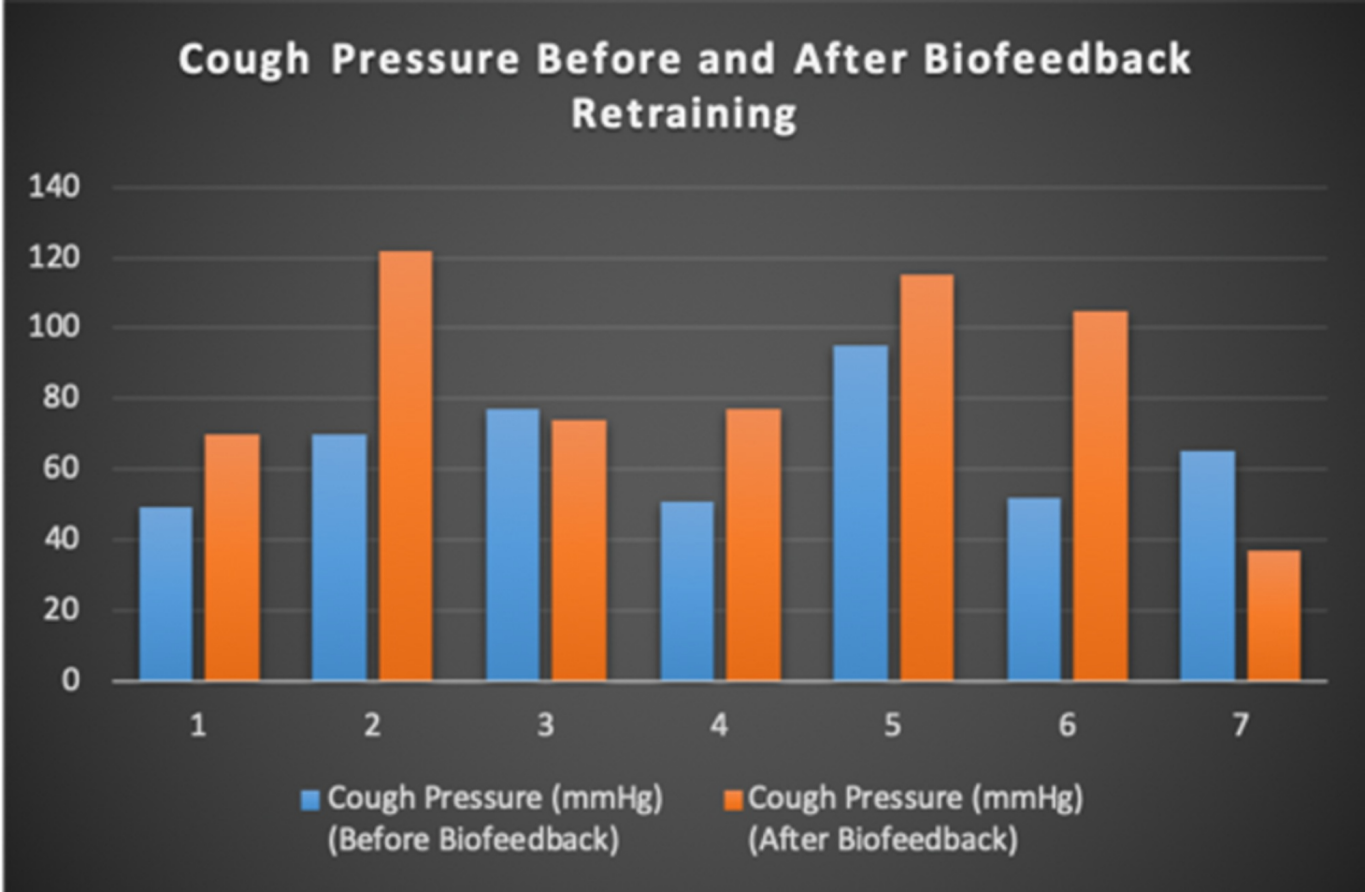
Cough Pressure For Each Patient Before and After Biofeedback Retraining

**Figure 10:**
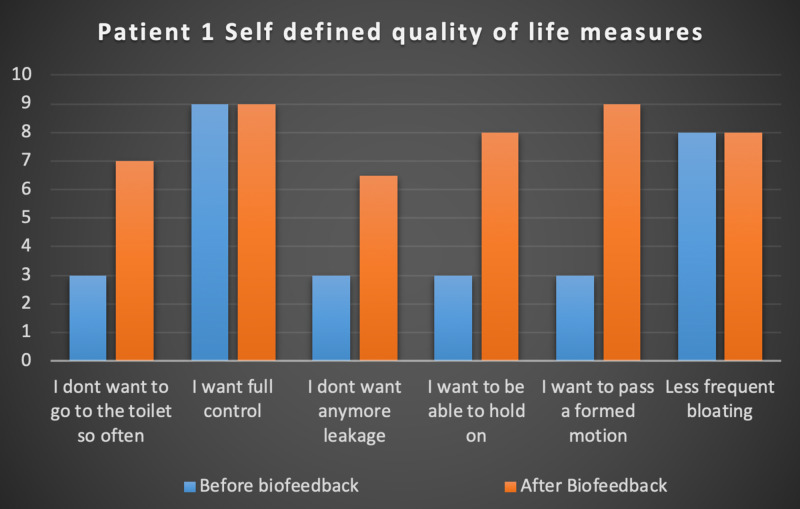
Patient 1 Self defined quality of life measures

**Figure 11:**
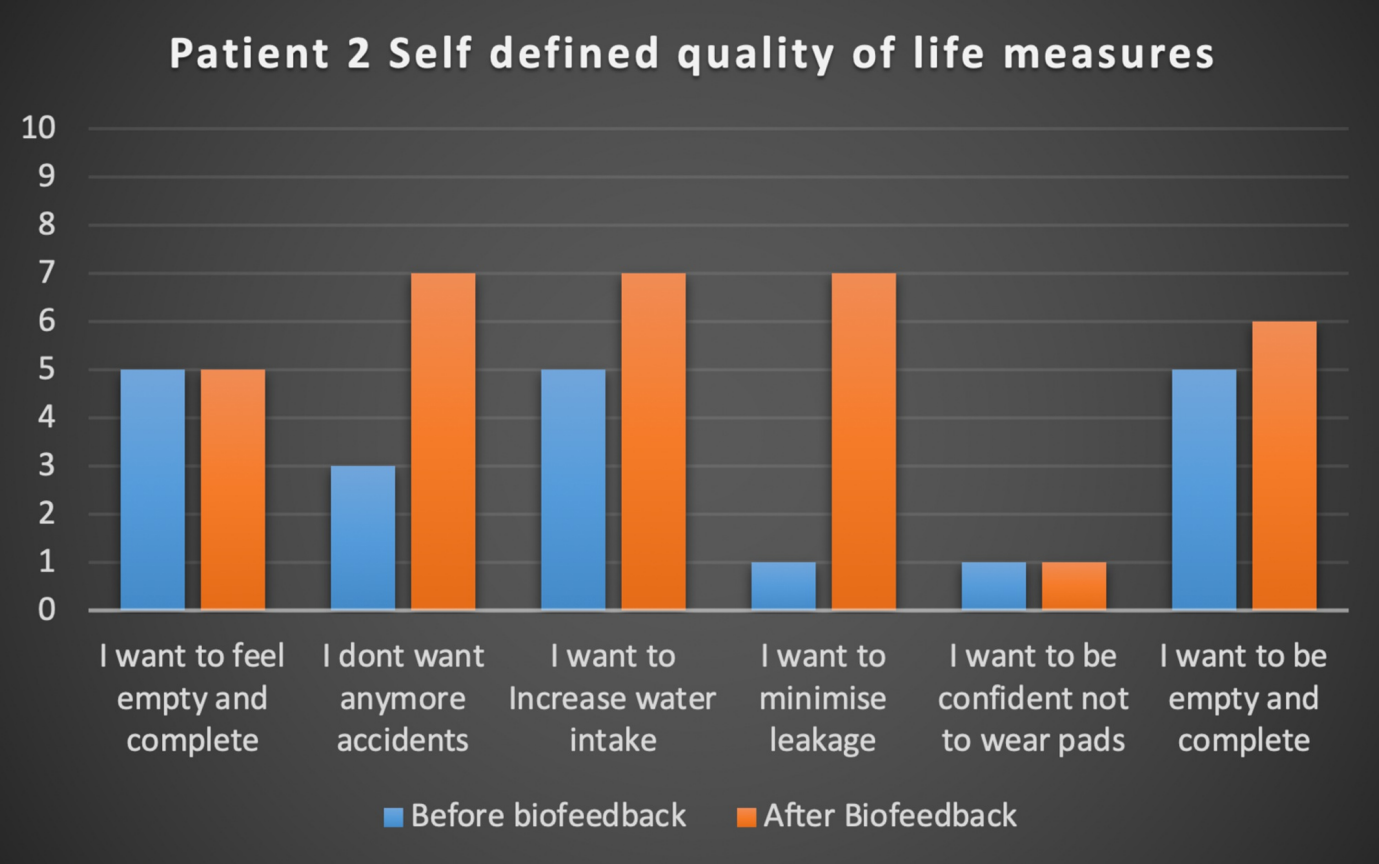
Patient 2 Self defined quality of life measures

**Figure 12:**
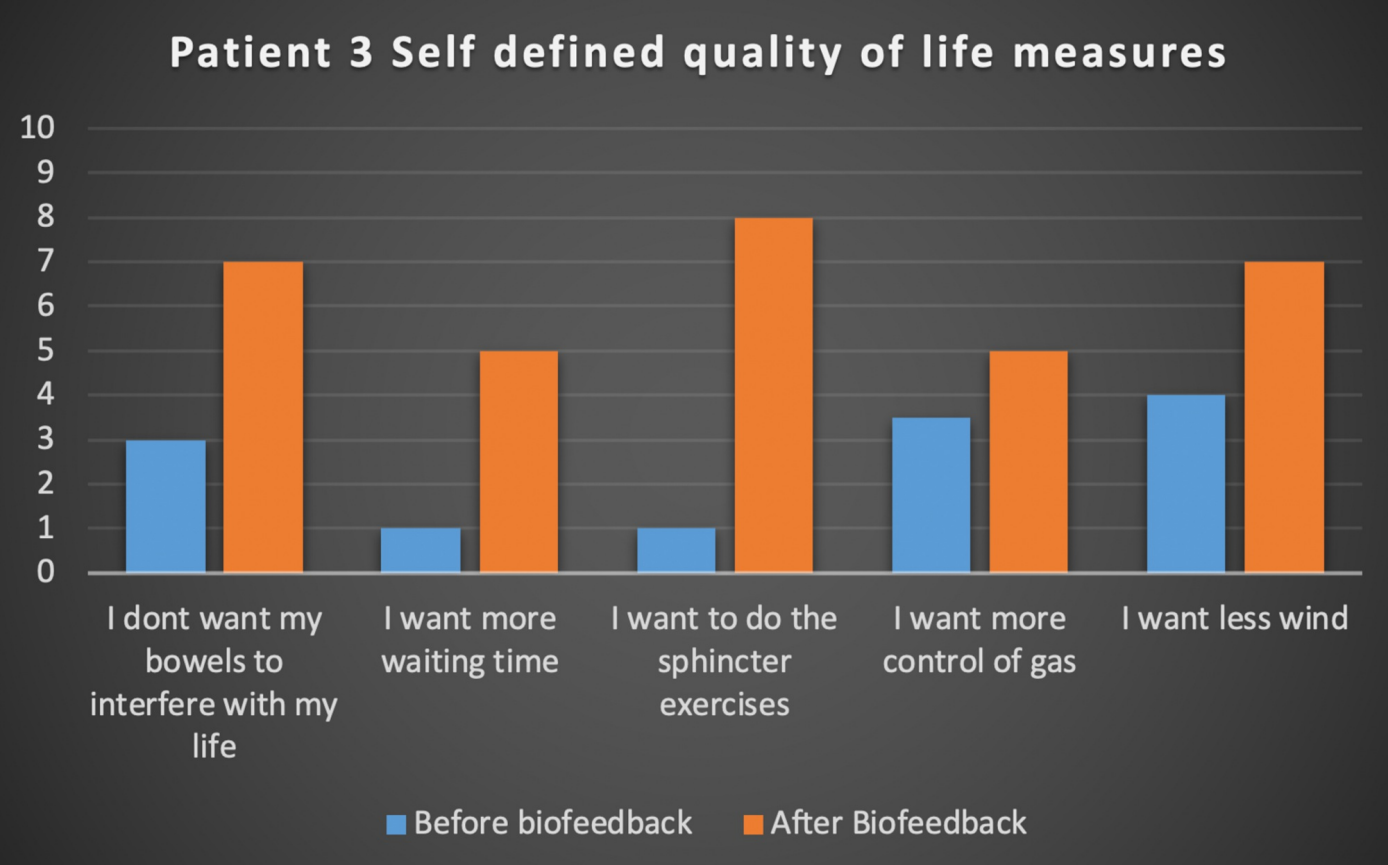
Patient 3 Self defined quality of life measures

**Figure 13:**
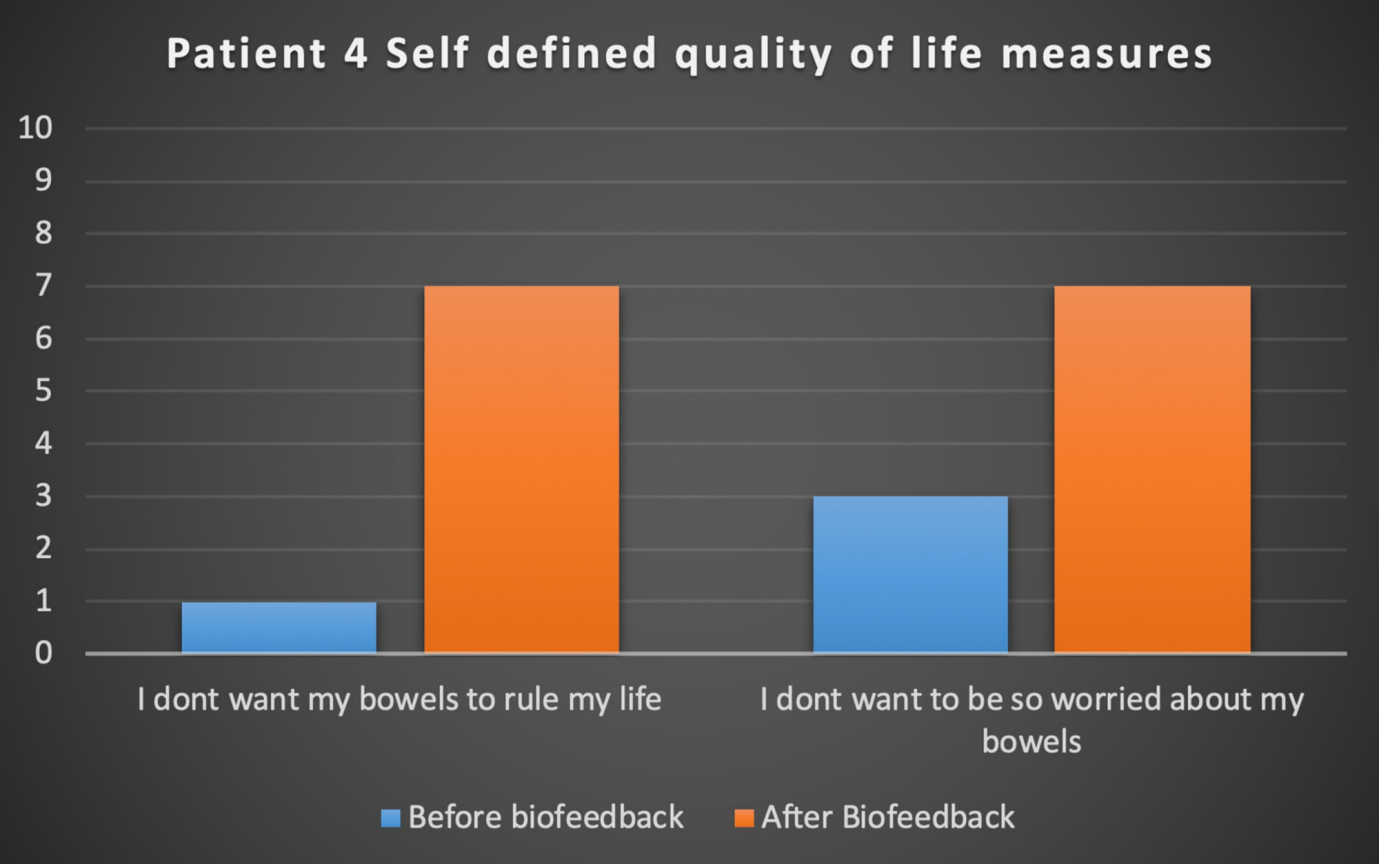
Patient 4 Self defined quality of life measures

Seven patients with radiotherapy-related faecal incontinence were identified. The radiotherapy target varied from prostate (4/7), uterus (2/7) and rectum (1/7). Two patients underwent a hysterectomy and one had an ultralow anterior resection. Three patients were male and four were female (57%). Pescatori (0-10) and St Mark’s (0-10) continence scores had a median improvement of 2 (range: 0-5 p<0.001) and 1 (range: 0-8 p<0.001) respectively (Figures 2, 3). Self-scored patient continence scores (0-10mm on a visual analogue scale) had a median improvement of 2 (range: 1-6mm p<0.001) (Figure 4). Anal fatigue time (Isotonic Fatigue Time (seconds) and Isometric Fatigue Time (Cycles)) showed a median increase of three seconds (range: -4 - 36 second p=0.10-0.20) and three seconds (Range: -1 - 6 seconds p=0.05 - 0.10) respectively (Figures 5, 6). Resting Pressure (mmHg), Squeeze Pressure (mmHg) and Cough Pressure (mmHg) showed median increase of 10mmHg (range: -10-21mmHg p>0.2), 15mmHg (range: -16 - 100mmHg p=0.10 - 0.20) and 21mmHg (range: -3 - 53mmHg p>0.2) respectively (Figures 7, 8, 9) but these differences were not statistically significant. Patient defined quality of life (QOL) measures showed a median improvement of 0.2 (range: 0-0.5 p<0.001). These measures were defined by the patient prior to starting treatment and included entries such as "I don't want any more leakage", "I want to be able to hold on" and "I don't want any more accidents". The patient then assigned a score between 0 (poor performance) to 10 (excellent performance). Four patients completed these at the beginning and end of biofeedback retraining and are shown in Figures 10-13.

## Discussion

This pilot study suggests an improvement in anal pressure metrics, continence, and QOL in patients who performed biofeedback-based anal strengthening exercises for radiotherapy-related faecal incontinence. This may be related to strengthening the sphincter complex which results in improved resting tone and continence in this patient group. Statistical significance was seen in Pescatori and St Marks continence scores, patient self-scored continence scores, and self-defined quality of life measures. There was a wide range of improvements in anal pressure measurements however these did not show statistical significance. Interestingly, the patient who underwent the ultralow anterior resection did show some improvement in continence scores but worsening anal pressure measurement (resting pressure, squeeze pressure, and cough pressure) and no improvement in the quality of life. This may be due to the proximity of the surgical procedure to the anal sphincter complex. 

We would argue that the continence scores and quality of life measures are important patient-perceived outcomes, perhaps more relevant than the objective measures obtained from the manometer. Patients reported improvement in key areas such as "reducing leakage", "decreasing accidents" and improving "waiting time". Patients generally have a keen interest in maintaining faecal continence and their engagement enables accurate assessment of their own function. 

The major limitation of this study is the small sample size. Although we included all patients that have undergone this treatment, the study still has a small heterogeneous group. We will address this shortfall in our future prospective study. There were three patients identified during the data collection phase that had not completed follow-up, so these patients were unable to be included in the study. 

Another limitation is that the biofeedback program we deliver is coordinated by an unblinded single nurse practitioner. This may introduce an unintended bias in the way in which the patient data has been initially collected. This was unavoidable with the design of the study however it would have been minimised by the use of our standardised data collection forms. 

An integral part of our retraining program is the inclusion of dietary advice around fibre and water intake, exercise guidance, education around functional toileting and psychological support. This may indicate that anal strengthening is not the sole contributor to continence improvement in our patient group however there is biological plausibility that a strong, intact anal sphincter would assist with maintaining continence in the patients. Studies suggest that biofeedback is effective however it is often delivered as a package of care rather than an isolated entity [16]. Many of these limitations will be addressed with a prospective randomised control trial.

## Conclusions

These early promising results suggest improved faecal continence in patients with radiotherapy related faecal incontinence and we are currently developing a prospective study to further evaluate the role of biofeedback training in this patient population.
